# Intrusive Thoughts Elicited by Direct Electrical Stimulation during Stereo-Electroencephalography

**DOI:** 10.3389/fneur.2016.00114

**Published:** 2016-07-18

**Authors:** Irina Popa, Cristian Donos, Andrei Barborica, Ioan Opris, Mihai Dragoş Mălîia, Mirela Ene, Jean Ciurea, Ioana Mîndruţă

**Affiliations:** ^1^Department of Neurology, University Emergency Hospital, Bucharest, Romania; ^2^Department of Physics, University of Bucharest, Bucharest, Romania; ^3^Epilepsy Center, University Hospital of Freiburg, Freiburg, Germany; ^4^FHC Inc., Bowdoin, ME, USA; ^5^Department of Neurosurgery, Bagdasar-Arseni Emergency Hospital, Bucharest, Romania; ^6^Department of Neurological Surgery, Miller School of Medicine, University of Miami, Miami, FL, USA; ^7^Department of Neurology, Carol Davila University of Medicine and Pharmacy, Bucharest, Romania

**Keywords:** epilepsy, forced thinking, prefrontal cortex, electrical stimulation, brain connectivity

## Abstract

Cortical direct electrical stimulation (DES) is a method of brain mapping used during invasive presurgical evaluation of patients with intractable epilepsy. Intellectual auras like intrusive thoughts, also known as forced thinking (FT), have been reported during frontal seizures. However, there are few reports on FT obtained during DES in frontal cortex. We report three cases in which we obtained intrusive thoughts while stimulating the dorsolateral prefrontal cortex and the white matter in the prefrontal region. In order to highlight the effective connectivity that might explain this clinical response, we have analyzed cortico-cortical potentials evoked by single pulse electrical stimulation.

## Introduction

Intrusive thoughts are a psychological phenomenon present in different neurological and psychiatric disorders. Understanding the structure and physiology of the brain networks eliciting this response will create new diagnostic and therapeutic opportunities for disorders such as schizophrenia, obsessive–compulsive disorder, or post-traumatic stress disorder (1).

The term forced thinking (FT) was first used by Penfield and Jasper, and it describes a rare ictal manifestation of frontal seizures. Since then, we found only a few reports where the authors have elicited “a little familiar thought” probably similar to a *déjà vu* state while stimulating the temporo-polar cortex (2). Given the scarcity of literature data on FT during direct electrical stimulation (DES) (2–4), the network involved is still a matter of research.

## Materials and Methods

We report three patients (P1–P3) with focal intractable epilepsy selected from a total of 52 who underwent invasive stereo-electroencephalography (SEEG) exploration at the Epilepsy Monitoring Unit of the University Emergency Hospital Bucharest, between February 2012 and March 2016. Patients provided written informed consent in accordance with the Declaration of Helsinki for the procedures that were part of the routine presurgical evaluation protocol. Medical records were used for patient and family history and neurological examination.

Phase I non-invasive pre-surgical evaluation included video-electroencephalography (VEEG) monitoring in order to record habitual seizures. Each patient also underwent 1.5- or 3-T magnetic resonance imaging (MRI) (isotropic 3D – sequence T1WI, axial and coronal FLAIR WI and T2 WI, T2* or SWI WI). Furthermore, P2 was explored with functional imaging (interictal FDG-PET scan). Invasive exploration was considered a necessary step in order to clearly delineate the epileptogenic zone and for functional cortex to be spared, as well as to establish the resection limits. Therefore, each patient underwent SEEG exploration using depth electrodes (Dixi Medical, Besancon, France), 8–18 contacts per electrode, 2 mm contact length, and 0.8 mm diameter, following an individual hypothesis. The electrodes were placed intracranially using the Leksell stereotactic frame (Elekta AB, Stockholm, Sweden). The post-implantation CT scan was coregistered with the pre-implantation MRI in the surgical planning software, in order to determine the exact location of each electrode and contact. Video-SEEG recordings were performed in chronic conditions at the University Emergency Hospital Bucharest in the Epilepsy Monitoring Unit using a 64-channel Nicolet Wireless Amplifier (Natus Neurology, Middleton, WI, USA), between 7 and 14 days. Stimulation was performed using the programmable stimulation unit of a Guideline 4000 LP+ system (FHC Inc., Bowdoin, ME, USA) Antiepileptic medication was tapered down when necessary to record seizures.

### Stimulation Procedure Protocol

Functional mapping of the cortex was systematically performed using DES. The stimulation protocol used frequencies of 1 Hz (biphasic, 3 ms pulse width, 40 s trains) and 50 Hz (biphasic, 1 ms pulse width, 5 s duration) on each pair of adjacent contacts in order to elicit electrographic or clinical responses, including ictal symptoms and signs. Multiple 5-s stimulation trials were performed, gradually increasing the current intensity from 0.1 to 3 mA, usually in 0.1 or 0.25 mA steps. Patients were instructed to report any psychological or physical changes that they experienced during or after each trial. In addition, patients were asked to perform different tasks during the stimulation session (e.g., counting, reading, word generation after a particular rule, object naming). The electrical stimulation was delivered 1–3 s after the task was initiated. The EEG was monitored for afterdischarges and seizure activity.

In order to highlight the relevant activity in specific frequency bands, associated with clinical responses during 50 Hz stimulation, we have created voltage maps, overlapped with patient’s scans. We have calculated the instantaneous amplitude of the EEG signal using the Hilbert transform. These maps were represented using color coding of the signal amplitudes, represented at the actual coordinates of the contacts on which they were recorded.

On the pairs of contacts located in the white matter in P2, eliciting clinical effects as a result of the 1- and 50-Hz stimulation, single pulse electrical stimulation (SPES) was performed to identify effective connectivity with cortical structures that were sampled (5). Evoked potentials were recorded on 62 out of 64 intracranial EEG channels.

For P2, SPES with variable pulse amplitudes [20 pulses per trial, 15 s inter-stimulus interval, 3 ms pulse duration, 0.25–5 mA stimulation current, varied in 0.25 mA steps, according to Donos et al. (6)], was applied on two pairs of contacts (Y′03-04 and Y′04-05) located in the white matter underlying the prefrontal cortex (PFC). For the stimulated areas, we have computed the effective connectivity, a specific form of functional connectivity that takes into account the directionality of the connections, using the method described by Donos et al. (7). The activation of various regions was shown as a color-coded map of the early response amplitude, overlapped with the patient’s MRI scans.

## Results

**Figure 1:**
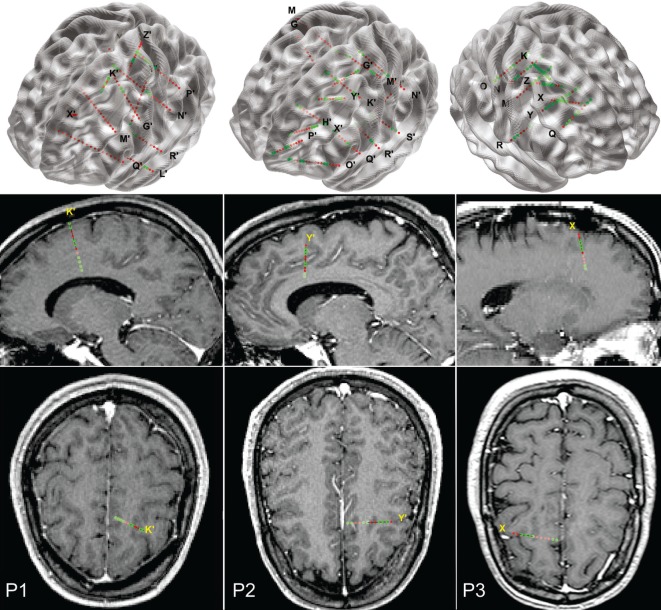
**Implantation charts for the three patients reported**. Top row, a 3D view of the electrode locations in the stereotactic space, superimposed with a MNI152 glass brain (aligned with the AC–PC line of each patient). Middle and bottom rows: sagittal and axial views of the electrodes, superimposed on actual patient’s MRIs.

**Figure 2:**
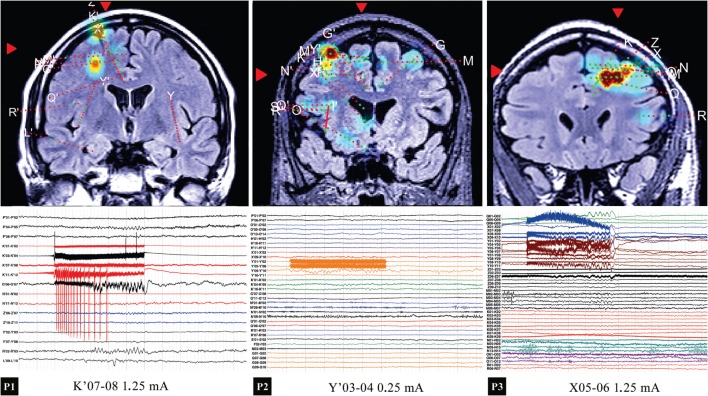
**Top row: location of the stimulated contacts for the three patients, with the instantaneous amplitude in the lower gamma band 30–45 Hz (excluding the stimulation artifact) shown as color map**. Bottom row: intracranial EEG of the 50-Hz stimulation. In P1, a local activation (contacts adjacent to K′07-08) was noticed, along with an activation in G′06-07. In P2, no significant responses were evoked, the high activity region visible in the map corresponding to the spontaneous activity in the hand area (M′09-10). In P3, an extended activation of the PFC is observed.

P1, a 35-year-old right-handed female, started having seizures at the age of 10 years. Her seizures were nocturnal and presented with a semiology, interictal, and ictal VEEG patterns suggestive for a left frontal epilepsy (Table 1). Patient’s 3-T MRI was negative. She underwent invasive exploration with 12 depth electrodes, 135 contacts according to a left mesial frontal hypothesis based on the scalp VEEG data (Table 1). We targeted key structures as dorsomedial prefrontal cortex (DMPFC), dorsolateral prefrontal cortex (DLPFC), middle cingulate cortex (MCC), presupplementary motor area (preSMA), supplementary motor area (SMA), premotor cortex (PMC), primary motor and sensitive cortex, rolandic operculum (OpR), amygdala, and middle temporal gyrus (MTG) on the left side and we also sampled the insula on each side with an oblique electrode (Figure 1). During the 50-Hz stimulation protocol, intrusive thoughts were obtained when stimulating electrode K′ contacts 07-08 located in DLPFC, in the sulcus between superior frontal gyrus and middle frontal gyrus (Mid-commissural point – MCP – coordinates – left: 16.44, anterior: 1.42, superior: 55.88) (Figure 2). The intensity of the current was 1.25 mA and no afterdischarges or other types of epileptiform activity were identified. Patient reported that “*the stimulation induces the disappearance of the word in my mind and replaces it with something else*.” She was not able to remember what that thought was. The effect lasted for 5 s, as long as the electrical stimulation was applied. Moreover, at an intensity of 3 mA, while performing a mental task (word generation in a semantic field), the patients stopped performing the task and reported again that “*the stimulation induces the disappearance of the word in my mind and replaces it with the idea of leaving*.” No afterdischarges were recorded, and the patient could restart the activity after the stimulation. This time, she also presented right head and eye version.

**Table 1:** **Patients included in the study and details regarding epilepsy and SEEG exploration**.

Patient	Sex	Seizure semiology	VEEG description	MRI lesion	Side	Electrodes	Contacts	Epileptogenic zone
P1	F	Aura: paresthesia propagated along the spine together with a warm sensation	*Interictal*: disorganized rhythm over the midline (Fz–Cz) with bi-frontal propagation of sharp waves	No	Left	12	135	MCC
		Ictal: grimacing, constriction inside the throat with fear of suffocation, rhythmic blinking, and hypermotor automatisms with secondary generalization when she was off medication	*Ictal*: fast activity involving the midline and the left frontal derivations followed by diffuse post-ictal flattening					
P2	M	Aura: none	*Interictal*: diffuse bi-frontal spikes and sharp waves more prominent over the midline and left prefrontal territories	No	Left	15	188	Prefrontal
		Ictal: grimace involving predominantly the upper part of the face rather than the lower part, followed by speech difficulty, head and eye version to the right, followed by secondary generalization	*Ictal*: flattening and baseline shift over the midline and left prefrontal regions followed by right central propagation and secondary generalization					
P3	M	Aura: fear	*Interictal*: polyspikes over the midline and right fronto-central regions with a phase reversal in F4	FCD type IIb	Right	9	112	PMC
		Ictal: left upper limb clonic movements, head and eye version to the left and secondary generalization	*Ictal*: baseline shift and fast activity in the midline followed by rhythmic theta discharge over the right central regions and left temporal, progressing to secondary generalization					

P2, a 28-year-old right-handed male, presented seizures since he was 11. Seizures semiology with grimace and speech difficulty in conjunction with scalp interictal and ictal activity suggested a left frontal epilepsy (Table 1). Cerebral MRI showed no lesion. He was therefore explored with 15 depth electrodes having 188 contacts, placed according to a left medial frontal hypothesis (Table 1). The electrodes targeted the frontal pole, mesial and lateral orbitofrontal cortex (MOFC, OFC), DMPFC, DLPFC, anterior cingulate cortex (ACC), MCC, preSMA, SMA, PMC, primary motor and sensitive cortex, suprasylvian operculum, and insula on the left side, together with MCC, PMC, and SMA on the right side (Figure 1). In P2, intrusive thoughts were obtained while stimulating electrode Y′ contacts 03-04 and 04-05 corresponding to the white matter in the prefrontal region (MCP coordinates: left: 15.44, anterior: 38.17, superior: 36.46) (Figure 2). We delivered multiple trials of stimulation, and the effect was elicited at variable intensities. During the first trial, the effect was elicited at 0.25 mA on Y′03-04. The patient reported that he had “*a though that seems to come from nowhere*.” In other trials of stimulation (Y′03-04, 3 mA), the patient reported a thought that he could not remember, but “*could be something embarrassing if said out loud*.” The patient was also asked to perform a mental task (counting from 1 to 100) that was interrupted during the 5 s of high frequency stimulation. When stimulating Y′04-05, we obtained the same effect at 1.75 mA.

**Figure 3:**
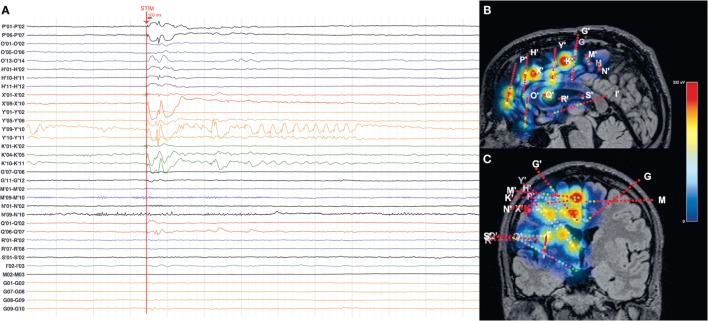
**(A)** Intracranial EEG illustrating the response to a single stimulation pulse in P2. **(B,C)** Maximum-intensity projections of the magnitude of the early responses on patient’s sagittal **(B)** and coronal **(C)** MRI views. Contacts that exhibited particularly high response to SPES stimulation were Y′03-04 and Y′04-05.

To evidence the network involved in eliciting intrusive thoughts, we have used the SPES protocol applied to Y′03-04 and Y′04-05 in P2, the site of DES-evoked intrusive thoughts. High-amplitude responses were evoked in a network involving ventromedial prefrontal cortex (VMPFC), DLPFC, DMPFC, PMC, preSMA, and dorsal-anterior insula, as shown in Figure 3.

P3, a 29-year-old right-handed male, started having seizures at the age of 17 years. He reports seizures with an aura consisting of fear and clonic upper limb movements that together with scalp EEG patterns were suggestive of a right frontal epilepsy (Table 1). Cerebral MRI revealed a type IIb focal cortical dysplasia of the PMC and DLPFC, at the bottom of the sulcus between the superior frontal gyrus and the middle frontal gyrus on the right hemisphere. The implantation hypothesis included the lesion and the anterior right frontal cortex, for functional mapping and establishing the resection limits (Table 1). We sampled the DMPFC, DLPFC, MCC, preSMA, SMA, PMC, primary motor and sensitive cortex, and OpR (Figure 1). In this case, intrusive thoughts were reported when stimulating at 1.25 mA in electrode X contacts 05-06 located in the middle frontal gyrus of the PFC (MCP coordinates: right: 17.29, anterior: 18.95, superior: 40.85) (Figure 2). Patient reported: “*I feel like someone erases all my thoughts and replaces them with something else that is capturing all my attention*.” These intrusive thoughts impair the stream of thinking; therefore, the patient ceases the word generation task.

## Discussion

Intrusive thoughts have been previously described as an ictal aura in frontal lobe epilepsy and in temporal lobe epilepsy. The latter ones usually are more intense and appear in conjunction with emotional symptoms due to the implication of the limbic system (8, 9). On the other hand, intrusive thoughts originating in the frontal lobe have a rational aspect and are associated with involvement of the language network (speech arrest) whenever the dominant hemisphere is involved (10). Previous reviews of frontal lobe epilepsy also mention intrusive thoughts accompanied by an attempt to act on the thought, as part of frontal lobe epilepsy (11). The fact that ictal intrusive thoughts are accompanied by other symptoms could be due to the ictal discharge recruitment of distant structures, in this case the cingulate cortex, described as part of the urge-to-action network (12). Our patients did not show any attempt of compulsive action while experiencing intrusive thoughts.

Consistent with the current knowledge, during frontal lobe stimulation, we were able to elicit intrusive thoughts invading patients’ mind and pushing away their own thoughts, with no emotional involvement. The responses were clearly different from auditory hallucinations or the inner speech phenomena described as verbal thinking (13). The stimulation was performed in DLPFC in P1 and in the white matter underlying the PFC mantle in P2 and P3. DLPFC is a neocortical structure with laminar–columnar organization (14) where each unit/module receives sensory input *via* bottom-up connections from the dorsal pathway (carrying spatial information) and/or *via* thalamo-cortical projections (15, 16) to the granular and deep layers of the frontal cortex. DLPFC integrates sensory inputs from virtually all cortical and subcortical structures (17) and plays a functional role in working memory and the executive control of behavior (18). Thus, DLPFC, in conjunction with the ACC, the preSMA, the inferior frontal junction, the anterior insular cortex, the dorsal premotor cortex, and the posterior parietal cortex plays an important role in thoughts control (19).

In P2 and P3, we have elicited intrusive thoughts when stimulating the white matter. This implies that we stimulated the axons of the cortico-striatal projections (15, 20) and/or the axons of the thalamo-cortical projections (21). Although we do not know the exact location of the projections on gray matter generating the effect, we do know that these axons are part of the cognitive thalamo-cortical loop (15, 21). Thus, the intrusive thought generated by the application of electrical current recruits different circuits than the “natural” thought that emerges in the absence of electrical stimulation. The low intensity of the stimulation current (0.25 and 1.25 mA, respectively) and the fact that other contacts stimulated at the same current intensities did not show similar responses point to some involvement of local activation of cortical modules that received mismatched feedback from the thalamic projections.

As revealed by the SPES protocol that we have applied in P2, most of these regions turned out to be functionally connected with the site of stimulation where we elicited intrusive thoughts. DLPFC projects to frontal eye fields that play a critical role in visual attention (22), in addition to eye movement control. Hence, the inhibition of task–performance that we found during stimulation could be due to the exogenous attention-shifting toward the intrusive thoughts. Indeed, this experimental observation is based on the fact that when more resources need to be devoted to inhibiting an action, less resources are available for the encoding/selection of sensory stimuli (23). Furthermore, PFC is connected with the premotor area, preSMA, SMA, and the rostral cingulate in order to plan and coordinate the behavioral action. Such frontal cortical connectivity could explain the behavior block during stimulation by taking into account the modulation of the executive output of DLPFC based on the behavioral context (17, 24).

Our results demonstrate that FT can be initiated by stimulating very well-defined regions of the PFC. A larger network, as evidenced by the connectivity analysis using cortico-cortical evoked potentials, may be involved in these behavioral and psychological manifestations.

## Author Contributions

All authors have contributed the same to the conception or design of the work or the acquisition, analysis, or interpretation of data for the work. IP, IM, and AB have highly contributed to drafting the work or revising it critically for important intellectual content. IM and AB have the major contribution in the final approval of the version to be published. All authors agree to be accountable for all aspects of the work in ensuring that questions related to the accuracy or integrity of any part of the work are appropriately investigated and resolved.

## Conflict of Interest Statement

The authors declare that the research was conducted in the absence of any commercial or financial relationships that could be construed as a potential conflict of interest.

## Abbreviations

ACC, anterior cingulate cortex; DLPFC, dorsolateral prefrontal cortex; DMPFC, dorsomedial prefrontal cortex; MCC, middle cingulate cortex; MCP, mid-commissural point; MOFC, mesial orbitofrontal cortex; MTG, middle temporal gyrus; OFC, orbitofrontal cortex; OpR, rolandic operculum; PMC, premotor cortex; PMC, premotor cortex; preSMA, presupplementary motor area; RMS, root mean square; SEEG, stereo-electroencephalography; SMA, supplementary motor area; SPES, single pulse electrical stimulation; VEEG, video-electroencephalography; VMPFC, ventromedial prefrontal cortex.
